# Identification of small ORF-encoded peptides in mouse serum

**DOI:** 10.1007/s41048-018-0048-0

**Published:** 2018-03-08

**Authors:** Yaqin Deng, Adekunle Toyin Bamigbade, Mirza Ahmed Hammad, Shimeng Xu, Pingsheng Liu

**Affiliations:** 1 National Laboratory of Biomacromolecules, CAS Center for Excellence in Biomacromolecules, Institute of Biophysics, Chinese Academy of Sciences, Beijing, 100101 China; 2 University of Chinese Academy of Sciences, Beijing, 100049 China

**Keywords:** Small ORF-encoded peptides (SEPs), Serum, Database, Mass spectrometric analysis, *ob/ob* mice

## Abstract

Identification of the coding elements in the genome is fundamental to interpret the development of living systems and species diversity. Small peptides (length < 100 amino acids) have played an important role in regulating the biological metabolism, but their identification has been limited by their size and abundance. Serum is the most important body fluid and is full of small peptides. In this study, we have established a small ORF-encoded peptides (SEPs) database from mouse GENCODE release. This database provides about half a million putative translated SEPs in mouse. We also extract serum proteins from wild type and *ob/ob* mice, and collect the low molecular weight proteins for mass spectrometric analysis. More than 50 novel SEPs have been discovered. Several SEPs are further verified by biochemical method with newly raised antibodies. These novel SEPs enhance the knowledge about the complexity of serum and provide new clues for the annotation and functional analysis of genes, especially the noncoding elements in the genome.

**Electronic supplementary material:**

The online version of this article (10.1007/s41048-018-0048-0) contains supplementary material, which is available to authorized users.

## Introduction

While biologists generally focus on the protein-coding open reading frame (ORF) of mRNA, it is now emerging that many mRNAs, even noncoding RNAs, also possess small ORF (sORF), and have significant roles in different organisms (Chu *et al.* 2015). It is a consensus that the ORFs in the transcribed mRNA will be translated into corresponding proteins due to in-frame codons defined by start and end codons. However, it still remains a big challenge in the field of gene annotation to distinguish the bona fide proteins from the translation noise. Moreover, most ORF-finding algorithms have historically set 300 nucleotides as the minimum ORF size for gene annotation, which incorrectly classifies genuine proteins corresponded RNA into noncoding RNAs (ncRNAs). On account to the great development of bioinformatics and biotechnology, numerous large-scale genomic studies have identified many nonclassical protein-coding genes, previous thought to be noncoding (Aramayo and Polymenis 2017; Bazzini *et al.* 2014; Chew *et al.* 2013; Derrien *et al.* 2012; Ingolia *et al.* 2011, 2014; Makarewich and Olson 2017; Tautz 2009; Ulitsky *et al.* 2011). More studies find that sORFs in ncRNA can encode small peptides, often referred as small ORF-encoded peptides (SEPs) that play important roles in the fundamental biological processes and in the maintenance of cellular homeostasis in different organisms, such as yeast, plant, zebra fish, *Drosophila*, and mammals (Anderson *et al.* 2015, 2016; Bazzini *et al.* 2014; Cohen 2014; Hanada *et al.* 2013; Ingolia *et al.* 2011; Ji *et al.* 2015; Lee *et al.* 2015; Magny *et al.* 2013; Matsumoto *et al.* 2017; Nelson *et al.* 2016; Smith *et al.* 2014).

Serum is the most important body fluid in mammals and possesses many important but low abundant small molecular proteins, such as peptide hormones, growth factors, lymphokines, and cytokines. However, few studies have revealed the existence and bioactivity of SEPs in serum. The major challenge in serum SEPs discovery arises from its extraordinary complexity in protein composition with the addition of post-translational modifications (PTMs) and protein variability, as well as the great concentration range (more than ten orders of magnitude) (Anderson and Anderson 2002; Omenn 2007).

To characterize the existence and bioactivity of SEPs in serum, we first established a mouse SEP database. This SEP database was then merged with mouse Uniprot database and Contamination database to form Mouse Merged database (MMD) for mass spectrometry (MS) data mining in this study. On the other hand, we extracted proteins with small molecular weight in different mouse sera and subjected to Q Exactive MS detection. After data mining, we discovered 54 novel SEPs in 15 serum samples. Furthermore, we raised four antibodies for four typical SEPs and finally confirmed the existence of two SEPs at the biochemical level.

## Results

### Construction and verification of Mouse Merged database

**Fig. 1:**
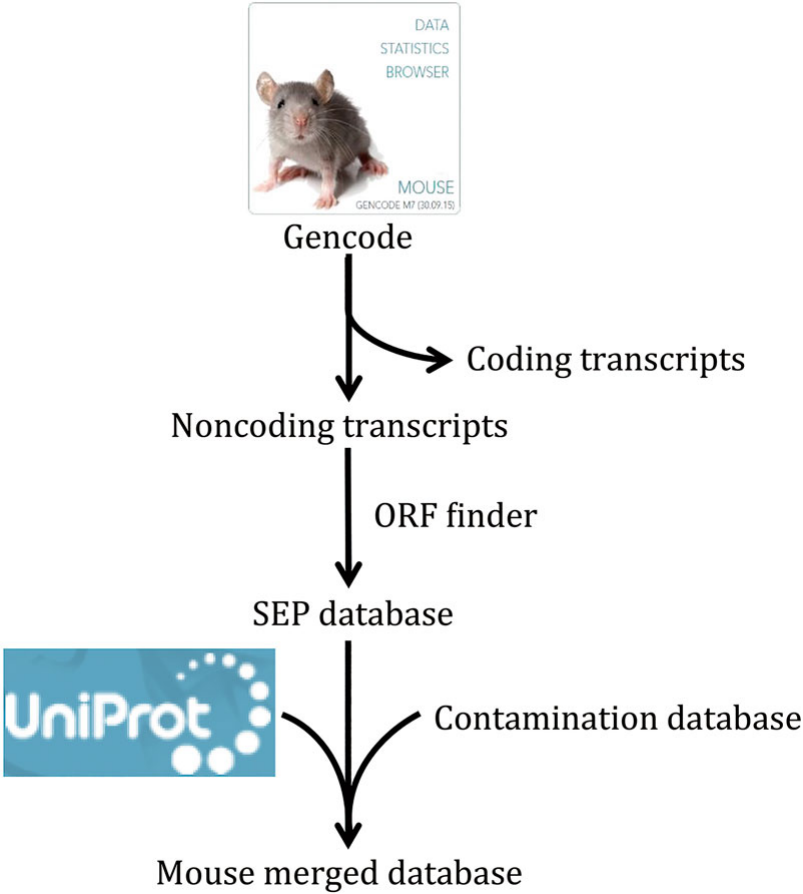
Construction of Mouse Merged database. Around 110 thousand transcripts of mouse were released from Gencode (vM4). All transcripts, except the known coding transcripts, were translated to SEPs (length between 8 and 100 a.a.) by the ORF Finder program and an in-house program. The SEP database was then merged with mouse Uniprot database and Contamination database to form Mouse Merged database for mass spectrometry data mining in this study

To characterize the existence of SEPs in serum, a novel mouse SEP database was constructed according to the RNA transcripts released from Gencode (vM4). This database provided about half a million putative translated SEPs in mouse. This database was then combined with mouse Uniprot database and Contamination database, forming MMD (Fig. 1). In order to verify the quality of the MMD, several recently identified functional SEPs were chosen and blasted within MMD. All of them could match one list in the MMD (Table 1). For example, MOTS-c is derived from a sORF in mitochondrial DNA and regulates insulin sensitivity and metabolic homeostasis (Lee *et al.* 2015). MLN, a conserved skeletal muscle-specific micropeptide, is derived from a sORF in a putative long noncoding RNA (lncRNA) and regulates skeletal muscle physiology (Anderson *et al.* 2015). SPAR is derived from a sORF in an lncRNA and inhibits muscle regeneration (Matsumoto *et al.* 2017). NoBody, a novel component of the mRNA decapping complex, is derived from a sORF in an lncRNA (D’Lima *et al.* 2017). Together, we have successfully constructed a high-quality MMD for the following MS data mining.

**Table 1:** Verification of the MMD

Accession	Sequence	Pub. SEPs	Reference
ENSMUSG00000064337.1	MKWEEMGYIFL	MOTS-c	Lee *et al.* (2015)
ENSMUSG00000019933.3	MSGKSWVLISTTSPQSLEDEILGRLLKILFVLFVDLMSIMYVVITS	MLN	Anderson *et al.* (2015)
ENSMUSG00000028475.8	METAVIGMVAVLFVITMAITCILCYFSYDSHTQDPERSSRRSFTVATFHQEASLFTGPALQSRPLPRPQNFWTVV	SPAR	Matsumoto *et al.* (2017)
ENSMUSG00000086316.3	MGDQPCASGRSTLPPGNTREPKPPKKRCVLAPRWDYPEGTPSGGSSTLPSAPPPASAGLKSHPPPPEK	NoBody	D’Lima *et al.* (2017)

### *ob/ob* mice show severe impaired glucose tolerance

**Fig. 2:**
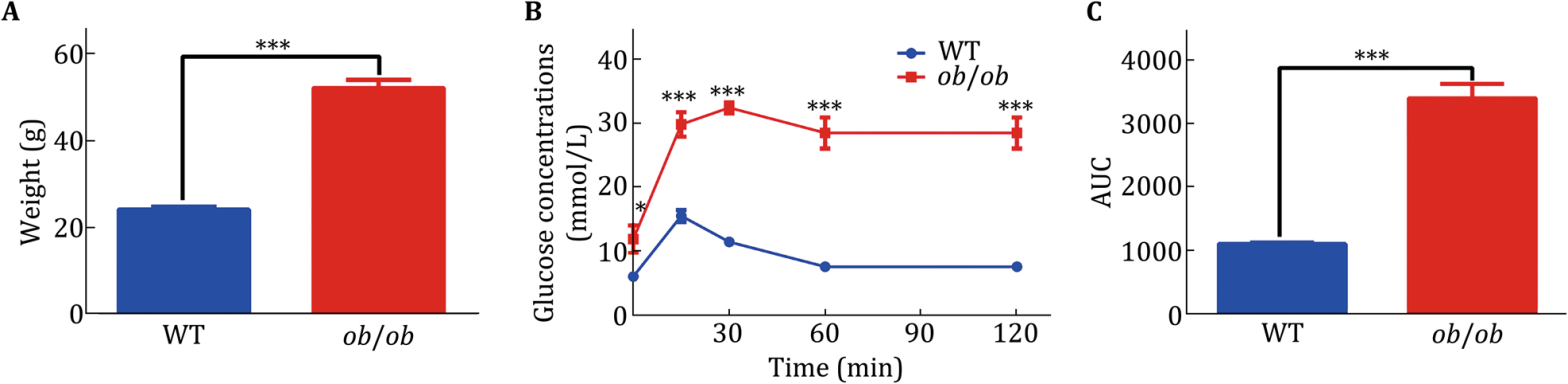
Verification of *ob/ob* mice. Twelve-week-old male mice were chosen for experiments in this study. **A** The weight of WT and *ob*/*ob* mice. **B** The IPGTT test for the WT and *ob*/*ob* mice. All the mice were fasting for 18 h before IPGTT. 2 g/kg glucose was injected for the IPGTT. **C** Area under the curve was calculated for the IPGTT. WT mice, *n* = 5; *ob/ob* mice, *n* = 6. Data were analyzed by Student *t* tests and presented as mean ± SEM. Significance, *p < 0.05; ***p < 0.001

As the species and the concentration of serum proteins show vast variability, wild-type (WT) and *ob/ob* mice were chosen for the serum protein preparation. We thought that some serum SEPs might show different expression patterns between WT and pathological mouse model. The body weight of *ob/ob* mice was significant higher than that of WT as indicated by previous studies (Fig. 2A). We then verified the glucose metabolism states of these two mouse models. *ob/ob* mice showed severely damaged glucose tolerance (Fig. 2B, C). These results show that these two typical mouse models possess dramatically different metabolic states.

**Fig. 3:**
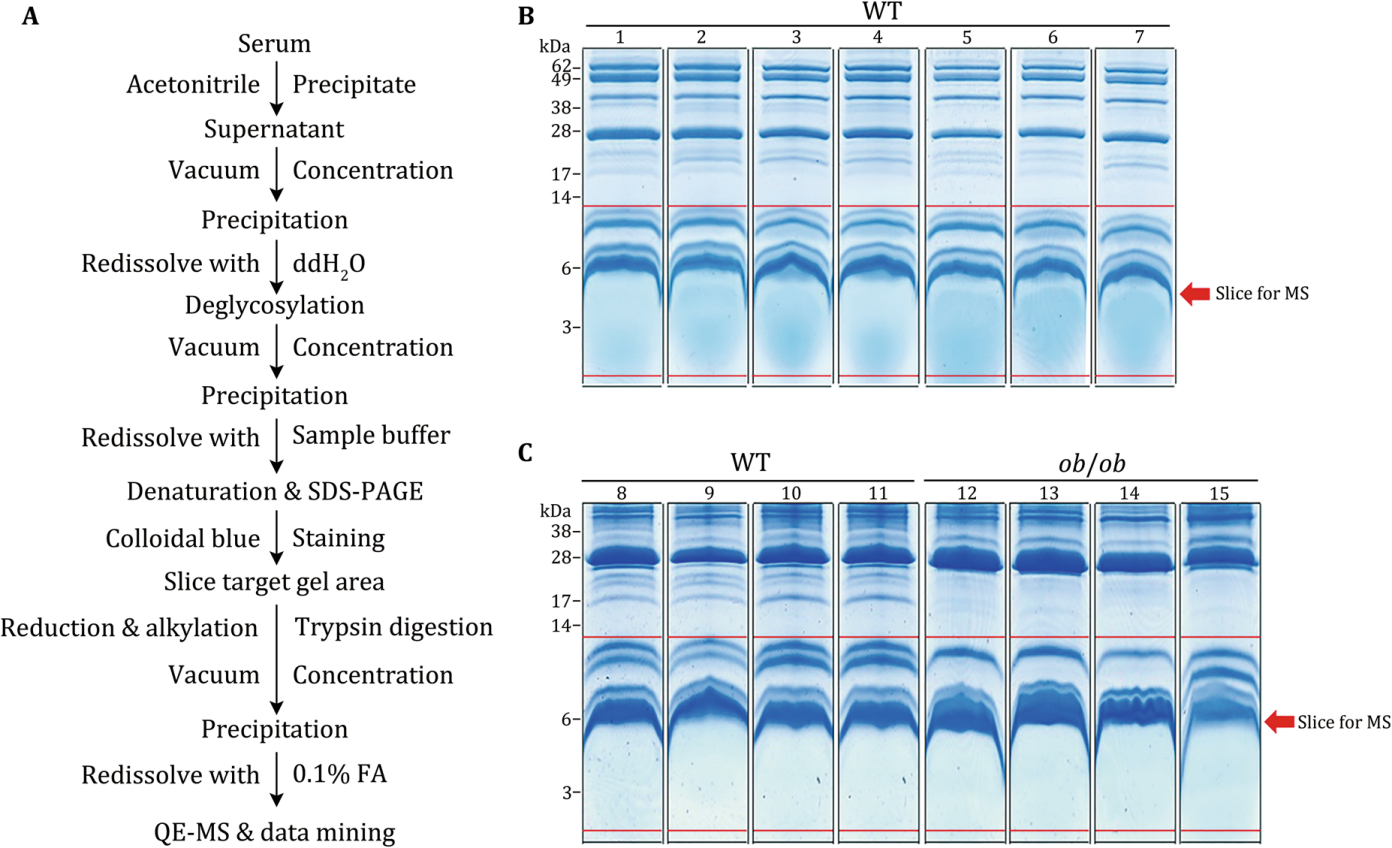
Working procedure for the serum SEP detection. According to the workflow for the enrichment and identification of low abundance mouse serum proteins (**A**), two rounds of mouse serum proteins were separated by SDS-PAGE and stained by Colloidal blue. **B** The first round, seven WT mice. **C** The second round, four WT mice and four *ob/ob* mice. The proteins below 14 kDa (the proteins between two *red lines* in every lane) were sliced for mass spectrometric analysis

We next extracted the serum proteins of WT and *ob/ob* mice according to the workflow showed in Fig. 3A. Since the putative serum SEPs might show very low abundance and high dynamics, 11 WT mouse samples and four *ob/ob* mouse samples were chosen for the following MS detection, respectively (Fig. 3B, C). As shown in Fig. 3B and C, all the serum samples showed clear protein staining signal in the low molecular weight range. We then sliced the gel area below 14 kDa for the following sample preparation and MS (the area between two red lines in every lane).

### MS detection discovers novel serum SEPs

**Fig. 4:**
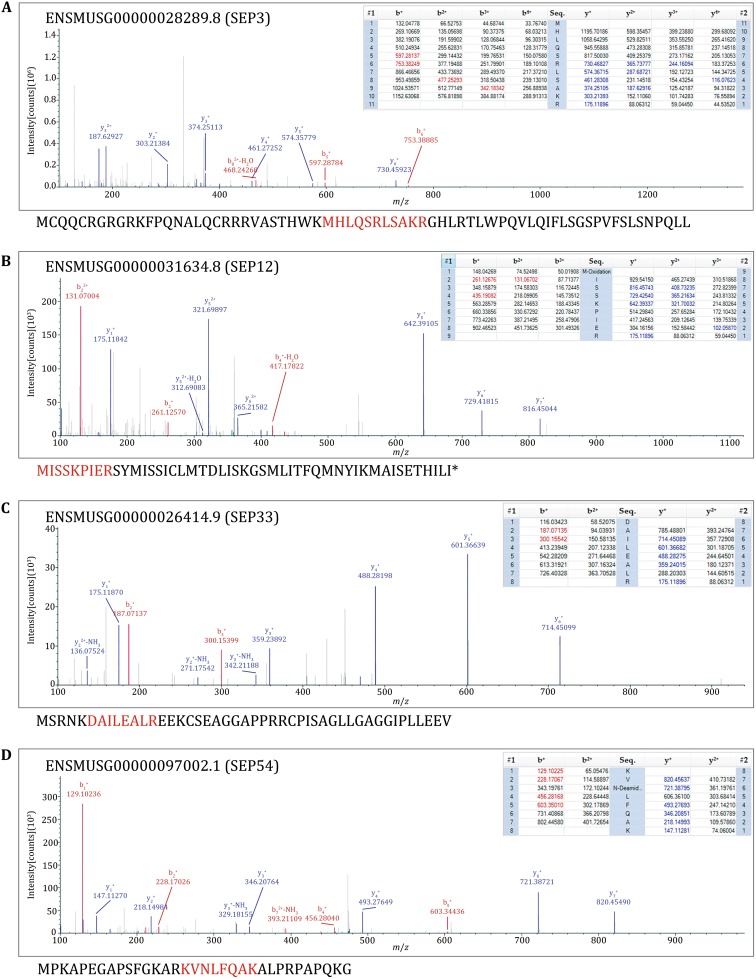
MS/MS spectrum of the four example peptides. The matched fragment ions of precursor ions were listed in the right of MS/MS spectra. All the matched ions were labeled with different colors, b-ions were labeled with *red color*, y-ions were labeled with *blue color*. The sequences below the spectra were the corresponding full length SEPs according to the Mouse Merged database. *Red highlights* represent the detected peptide fragments. **A** The spectrum result of SEP3. **B** The spectrum result of SEP12. **C** The spectrum result of SEP33. **D** The spectrum result of SEP54

**Fig. 5:**
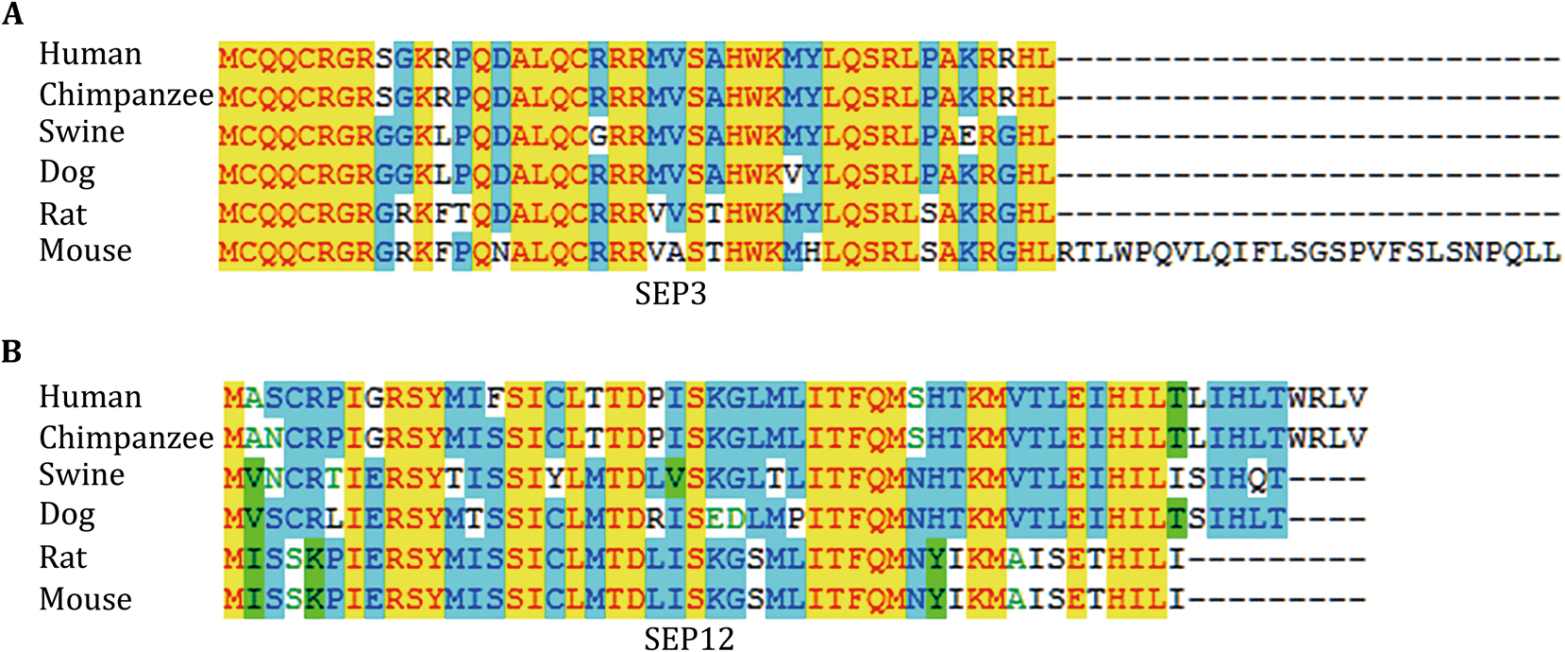
SEP3 and SEP12 are conserved in mammals. Conservation analysis of SEP3 (**A)** and SEP12 (**B)** with clustal multiple alignment in six species

The sliced gels were further processed for MS detection according to the workflow in Fig. 3A. 54 novel SEPs were detected in total from the 15 samples (Table 2). Eight SEPs were detected in more than one sample. 38 SEPs were only detected in WT mouse serum and 12 SEPs were only detected in *ob/ob* mouse serum (Table 2). We sequentially named the SEPs from SEP1 to SEP54 (Table 2). SEP3, SEP12, SEP33, and SEP54 were chosen for further study to confirm the accuracy of Q Exactive MS results and to verify the existence of the SEPs in serum. The MS/MS spectrums of these four SEPs were presented in Fig. 4. SEP3 was detected in Sample 1 and Sample 2, and was encoded from a sORF in processed transcript of *Epha7* gene (Table 2, Fig. 4A). Besides, SEP3 was conserved in mammals (Fig. 5A). SEP12 was detected in five samples and was encoded from a sORF in processed_transcript of *Ufsp2* gene (Table 2, Fig. 4B). SEP12 was also conserved in mammals (Fig. 5A). SEP33 was detected in four samples and was encoded from a sORF in retained_intron of *Tnnt2* gene (Table 2, Fig. 4C). SEP54 was detected only once with high X correlation score in Sample 15 and was encoded from a sORF in lncRNA Gm2670 (Table 2, Fig. 4D). All of the four primary MS results strongly suggested the detection of targeted peptides. Taken together, these lines of evidence suggest that SEPs widely exist in the serum and might show wide individual differences.

**Table 2:** MS identification of sliced bands

Band NO.	SEP NO.	Accession	Description	Detected peptide	XCorr	M.W. (kDa)
1	1	ENSMUSG00000032985.11	5730522E02Rik|processed_transcript	LPQLAAAEPNRPR	2.22	10.6
	2	ENSMUSG00000085596.1	Gm11476| processed_transcript	QLPLYQIEILVcNITAmTHPSNFSlESNQcRLSPRSQQLEcPK	2.11	8.8
	3	ENSMUSG00000028289.8	Epha7| processed_transcript	MHLQSRLSAKR	1.65	8.0
	4	ENSMUSG00000029049.10	Morn1 | processed_transcript	NGTGcVSPLELR	1.09	6.4
	5	ENSMUSG00000021177.11	Tdp1|retained_intron	MVcLSFTTK	0.75	1.4
	6	ENSMUSG00000042105.14	Inpp5f|retained_intron	MLVLPVnLPR	1.73	1.3
	7	ENSMUSG00000029647.11	Pan3|nonsense_mediated_decay	MLLIcNQKQMHLPSSWPTSSDR	1.47	2.7
	8	ENSMUSG00000081985.1	lincRNA 1700047M11Rik|lincRNA	SNRSLTLR	1.05	1.4
	9	ENSMUSG00000081985.1	Gng2-ps1|processed_pseudogeiie	KLIELLKmEAnlDR	1.05	6.0
	10	ENSMUSG00000006423.11	C330007P06Rik|retained_intron	HPPVIFVTYTHMQANTHAHKIR	1.36	6.3
	11	ENSMUSG00000059974.6	Ntm|processed_transcript	TTQAKMHnSISWAIFTGLAALcLFQGK	1.49	3.2
2	3	ENSMUSG00000028289.8	Epha7|processed_transcript	MHLQSRLSAKR	1.65	8.0
	12	ENSMUSG00000031634.8	Ufsp2|processed_transcript	mISSKPIER	1.66	2.8
	1	ENSMUSG00000032985.11	5730522E02Rik|procssed_transcript	LPQLAAAEPNRPR	1.60	10.6
3	13	ENSMUSG00000054693.10	Adam10|nonsense_mediated_decay	KEALVmGLSLMEDLKVSSR	0.90	9.6
	14	ENSMUSG00000035953.9	Tmem55b|retained_intron	HFPRSLRDIQPccLER	1.28	3.4
	15	ENSMUSG000000103242.1	RP23-132G24.3|processed_pseudogene	MAEAIYIIEVKEWGK	1.33	2.5
4	16	ENSMUSG00000085553.1	antisense Gm14808|antisense	QnNHGGWLWVPKEScALGR	1.65	7.4
	12	ENSMUSG00000031634.8	Ufsp2|processed_transcript	mISSKPIER	1.65	2.8
	17	ENSMUSG00000048215.10	lincRNA A630023P12Rik|lincRNA	SQNFSWImLLcPSQM	0.47	4.9
	18	ENSMUSG00000086528.1	antisense Gm15731|antisense	TIQKAPPHYmSIELR	1.60	4.1
	19	ENSMUSG00000102503.1	TEC RP23-388I22.1|TEC	mYYLVKmScYmKcLR	0.32	2.7
5	20	ENSMUSG00000085865.1	lincRNA Gm15966|lincRNA	SASSWNQPLPGPSGFGLEEVSREGGWR	3.36	5.8
	21	ENSMUSG00000081123.1	Gm11469|processed_pseudogene	EGVNIAEAIER	1.60	10.1
	4	ENSMUSG00000029049.10	Morn1|processed_transcript	NGTGcVSPLELR	1.45	6.4
	22	ENSMUSG00000053199.9	Arhgap20|retained_intron	QSTVKcWRPFQmSHmQTFmK	1.15	7.7
	23	ENSMUSG00000029464.6	Gpn3|retained_intron	PGGAERnSR	0.78	2.4
	24	ENSMUSG00000098033.1	Gm9381|processed_pseudogene	IVSnAScTTncIVLLAKVIFGmTTLALER	1.69	9.3
	25	ENSMUSG00000102240.1	TEC RP23-242B14.1|TEC	mNLKILTYVcFASQRQTIYLENR	2.21	5.5
	26	ENSMUSG00000020063.12	Sirt1|nonsense_mediated_decay	mVFHTFLFVTLnSLK	1.06	3.1
	11	ENSMUSG00000059974.6	Ntm|processed_transcript	TIQAKMHnSISWAIFTGLAALcLFQGK	2.30	3.2
6	27	ENSMUSG00000024073.10	Birc6|retained_intron	QLFLVEnKNLNIIIPmFYcFFPIR	1.22	11.3
	28	ENSMUSG00000057406.12	Whsc1|nonsense_mediated_decay	SLPSQKcSPKYSENEAR	0.83	3.9
	29	ENSMUSG00000031559.10	4930555F03Rik|processed_transcript	mLHcVHSSLIYnSQTLER	1.58	4.9
7	30	ENSMUSG00000072929.6	Gm15109|unprocessed_pseudogene	DTMVQEEEMDQGMHHHQDLSQK	0.24	3.9
	31	ENSMUSG00000090699.1	Gm9071|unprocessed_pseudogene	mKEKEVMSFLHNLEMEYIEAR	1.51	6.2
	32	ENSMUSG00000025495.10	Ptdss2|nonsense_mediated_decay	NPSGYSLQHQERYcGQYFGFLMFWSHT	1.01	6.9
8	33	ENSMUSG00000026414.9	Tnnt2|retained_intron	DAILEALR	1.67	4.8
	34	ENSMUSG00000025089.11	Gfral|nonsense_mediated_decay	FPHTFYHRVLIcSTAWDPNK	1.12	7.0
9	35	ENSMUSG00000034285.11	Nipsnap1|nonsense_mediated_decay	IEVLGSLFR	1.97	6.6
	33	ENSMUSG00000026414.9	Tnnt2|retained_intron	DAILEALR	1.61	4.8
	36	ENSMUSG00000084274.2	Gm12504|processed_transcript	MNYFcFHImWcYVLSFmAR	0.33	3.7
	15	ENSMUSG00000103242.1	RP23-132G24.3|processed_pseudogene	MAEAIYIIEVKEWGK	1.06	2.5
	37	ENSMUSG00000102415.1	TEC RP23-284P20.1|TEC	LTKTYQHVYcMLK	0.91	3.2
10	33	ENSMUSG00000031626.12	Tnnt2|retained_intron	DAILEALR	1.72	4.8
	38	ENSMUSG00000031626.12	Pros1|retained_intron	EnmDSnHKKTVFSILLEMR	0.23	4.8
	39	ENSMUSG00000031626.12	Gm29365|unprocessed_pseudogene	SVTDmDTIEKSNLnRQFLFcPWDVTK	0.64	8.5
11	1	ENSMUSG00000032985.11	5730522E02Rik|processed_transcript	LPQLAAAEPNRPR	2.16	10.6
	40	ENSMUSG00000092054.3	Kif4-ps|transcribed_processed_pseudogene	mLTELEK	1.09	5.7
	41	ENSMUSG00000090109.1	Ear-ps10|unprocessed_pseudogene	TTVAMKSYTVAcNPR	1.50	7.9
	42	ENSMUSG00000099956.1	Gm29365|unprocessed_pseudogene	DPAFYAYQLLDDYKEGnLHMIPDTPPAEERSGDDSDVLIGn	0.61	6.0
12	43	ENSMUSG00000031626.12	Sorbs2|processed_transcript	YQIFnFnR	1.70	2.4
	44	ENSMUSG00000022686.10	B3gnt5|processed_transcript	FVLETFPPGLLGGQRTSGTFK	1.10	4.5
	45	ENSMUSG00000083128.1	Gm12723|processed_pseudogene	TEAIEALVK	1.18	5.7
	12	ENSMUSG00000031634.8	Ufsp2|processed_transcript	mISSKPIER	1.82	2.8
	46	ENSMUSG00000020361.9	Hspa4|processed_transcript	TQYVDHAGLELKGSHQPLPPK	1.03	5.2
	47	ENSMUSG00000091078.1	antisense Gm17218|antisense	MASVSPEIKR	1.38	1.6
13	12	ENSMUSG00000031634.8	Ufsp2|processed_transcript	mISSKPIER	1.88	2.8
	48	ENSMUSG00000103862.1	TEC RP23-198F7.2|TEC	mRNWLVSPmnSK	1.24	4.1
14	6	ENSMUSG00000042105.14	Inpp5f|retained_intron	MLVLPVnLPR	2.34	1.3
	1	ENSMUSG00000032985.11	5730522E02Rik|processed_transcript	LPQLAAAEPNRPR	1.85	10.6
	49	ENSMUSG00000042688.12	Mapk6|retained_intron	FLFTnR	1.65	1.7
	12	ENSMUSG00000031634.8	Ufsp2|processed_transcript	mISSKPIER	1.58	2.8
	50	ENSMUSG00000076594.1	Ikgv6-13|IG_LV_gene	ASQn	1.30	10.1
	51	ENSMUSG00000005360.10	Slcla3|retained_intron	VWEAPRYnK	1.40	6.2
	52	ENSMUSG00000103591.1	TEC RP24-369B15.2|TEC	mMLKTIcRIINVFLILLnEDDAK	1.26	5.4
	53	ENSMUSG00000006010.10	BC003331 |retained_ intron	ELSWIIWmKNGPQNMPAR	1.48	3.0
15	54	ENSMUSG00000097002.1	lincRNA Gm2670|lincRNA	KVnLFQAK	1.98	3.5
	33	ENSMUSG00000026414.9	Tnnt2|retained intron	DAILEALR	1.93	4.8

Mouse Merged database was used for mass spectrometry database search

### Western blot results confirm the existence of serum SEPs

**Fig. 6:**
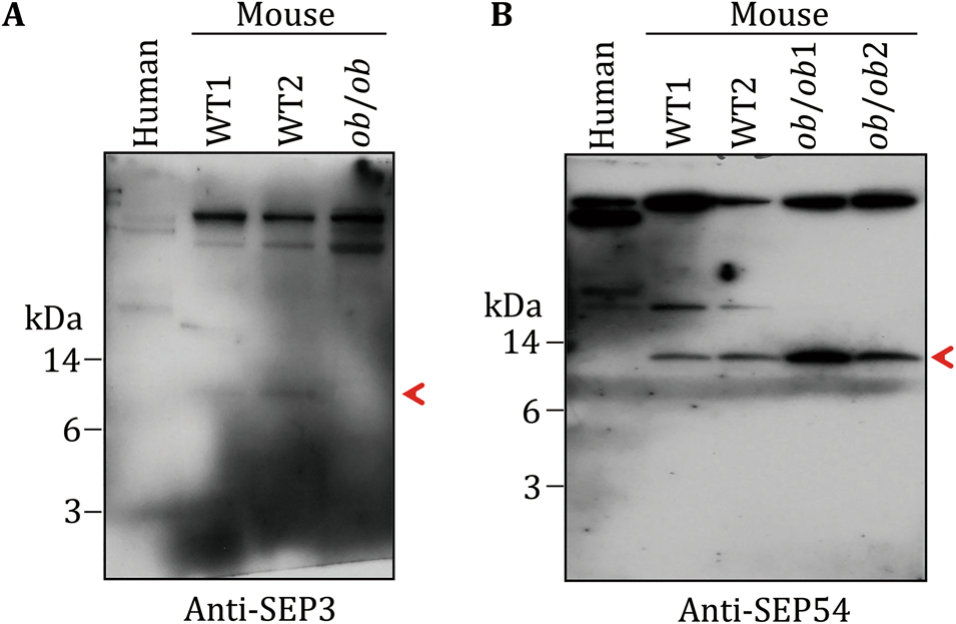
WB verification of SEPs in mouse serum. Polyclonal antibodies for four SEPs were raised in rabbits. Two antibodies showed specific bands in the low molecular weight area of mouse serum samples. **A** Anti-SEP3 antibody recognized a target protein in around 8 kDa, indicated by the *red arrow*. **B** Anti-SEP54 antibody recognized a target protein in around 10 kDa, indicated by the *red arrow*

In order to further confirm the existence of the SEPs, four antibodies were raised to against SEP3, SEP12, SEP33, and SEP54. The antigens were designed as indicated in materials and methods. The sera from the immunized rabbits were used as the antibodies to detect the corresponding SEPs in mouse sera by Western blot with human serum as control. Consistent with the MS results, SEP3 antibody recognized an 8-kDa protein in WT mouse serum (Fig. 6A), rather than that of human and *ob/ob* mouse. Similarly, SEP54 antibody recognized a 10-kDa protein in mouse serum (Fig. 6B), rather than that of human. Furthermore, consistent with the MS result, SEP54 showed higher concentration in *ob/ob* mouse. However, SEP12 and SEP33 antibodies failed to recognize any specific band in all of the serum samples (data not shown). Altogether, these results further demonstrate the existence of SEPs in serum with different expression levels.

## Discussion

In this study, we constructed a novel SEP database and discovered some SEPs in serum by MS. Our data provided two key insights into the genome-wide expression of SEPs in mammals. First, SEPs were widely distributed and translated from a large body of transcripts. We annotated hundreds of thousands of SEPs (length ranging from 8 to 100 a.a.) according to the noncoding transcripts in mouse GENCODE (vM4), and validated 54 novel SEPs in the mouse serum (Table 2). This was the first systematic study to explore the existence of SEPs in serum. Previous studies have successfully used computational approach and ribosome profiling to define the transcripts in translating ribosome (Bazzini *et al.* 2014; Chew *et al.* 2013; Ingolia *et al.* 2009, 2011; Menschaert *et al.* 2013) and identified some SEPs in specific tissues and cell lines. However, it has been strongly argued whether the RNA fragments protected by the ribosome always reflect the actively translated transcripts. The RNA bound to RNA-binding proteins will also be improperly classified as coding sequence, as well as the ribosome randomly bound RNA. In consistent with the known serum small peptides, the putative serum SEPs might also be low abundant, highly dynamic, and low molecular weight. Therefore, a high-precision mass spectrometer and a homemade database were chosen to verify the existence of SEPs in the serum.

Second, the serum SEPs were very low abundant and highly dynamic among individuals. For example, ApoA2, a well-known high abundant serum peptide (Uniprot: P09813; length: 102 a.a.), was detected more than ten fragments in every sample of all of the 15 samples (data not shown). However, all of the 54 SEPs detected in this study matched only one fragment in the corresponding sample (Table 2), and only eight SEPs were repeatedly detected in more than one sample (Table 2). Besides, the low abundance of those SEPs might also be one of the reasons for the weak Western blot signal of SEP3 and SEP54 (Fig. 6). On the other hand, insulin, a protein existing in serum with nanogram level, was not detected in the 15 samples (data not shown), which suggests that the abundance of the above-detected SEPs might be higher than that of insulin and further proved the existence of those SEPs in serum. Besides, the low repeatability detection of the above 54 SEPs among 15 samples from 11 WT mice and four *ob/ob* mice implied, to some degree, the high dynamics of serum SEPs among individuals (with different metabolic states). These MS results were further verified by Western bolt analysis. Consistently, SEP3 antibody detected stronger signal in WT mouse serum than that in *ob/ob* mouse serum, in agreement with the result that SEP3 was only detected in WT mouse samples in MS results. Similarly, SEP54 antibody detected stronger signal in *ob/ob* mouse serum samples than that in WT mouse sample. These high dynamics of SEPs were similar with that of known small peptides in serum. For example, serum insulin level increases after feeding and serum irisin level increases after exercise (Jedrychowski *et al.* 2015). Besides, signal peptide prediction showed only three of the detected SEPs had the secretion signal peptide (see Supplemental table), which indicated that most SEPs tended to be secreted by uncanonical pathway, or released from broken cells.

Several approaches have been used to validate the putative SEPs (Housman and Ulitsky 2016). Ideally, the generation of antibodies against target SEPs is the most effective method (Anderson *et al.* 2015). However, the optimal antigen designing to the SEPs is challenging for their small size. This may be the reason why antibodies raised by SEP12 and SEP33 could not recognize specific bands in serum samples. As antigen peptides for SEP3 possessed 4 a.a. difference between human and mouse, SEP3 antibody could not recognize its ortholog in human SEP3 (Figs. 5A, 6A). Another concern for the usage of antibody is that even the highest-affinity antibody may not be sufficient to produce a strong enough signal for the detection of low abundance SEPs. Alternatively, clustered regularly interspaced short palindromic repeats (CRISPR)-CRISPR associated protein 9 (Cas9) mediated gene-editing for the target SEPs *in vitro* or *in vivo* could also provide direct evidence for the existence of the SEPs and substantially support the functional study of the SEPs. Besides, for the biological function study of serum SEPs, such as SEP3 and SEP54, the mouse tail vein injection of artificially synthesized full length SEPs will be a high-efficiency approach.

It still remains unclear how many SEPs exist in serum and what the biological functions of the serum SEPs are. New methods and new ideas are still needed to further study the SEPs in serum. Together, our study opens a new avenue for the identification of small peptides in serum, and provides an entry point to investigate their function *in vivo*.

## Materials and methods

### Animals

Twelve-week-old male WT (C57BL/6J) and *ob/ob* mice were housed in our animal facility on a 12-h light/dark cycle with ad libitum access to water and food. All animal protocols were approved by the Animal Care and Use Committee of the Institute of Biophysics, Chinese Academy of Sciences, SYXK (SPF 2011-0029).

### IPGTT

Tail blood samples were collected from 12-week-old mice that had been fasted for 18 h, before and at 15, 30, 60, and 120 min after i.p. injection of glucose (2 g/kg). Glucose levels were measured at prespecified times. Blood glucose was measured using glucometer (ACCU-CHEK, Roche).

### Mouse Merged database construction

For the construction of SEP database, both the ORF Finder program and an in-house program were used to identify ORFs from noncoding transcripts in mouse GENCODE (vM4). Ensembl transcripts (release 73) were downloaded from the Ensembl FTP repository to annotate the noncoding transcripts in mouse GENCODE. The peptide sequences associated with predicted ORFs of noncoding RNAs, ranging from 8 to 100 a.a., were selected to construct the SEP database. The SEP database was then merged with mouse Uniprot database and Contamination database to form Mouse Merged database (Supplemental data).

### Serum sample preparation

The workflow for the preparation of serum samples was shown in Fig. 3A. Serum samples were collected from 12-week-old WT and *ob/ob* mice by removalling eyeballs. After clotting, serum was separated by centrifugation at 3000 *g* for 10 min at 4 °C. The low molecular weight and low abundance serum proteins were enriched with 60% acetonitrile as previous reported (Echan *et al.* 2005; Kay *et al.* 2008; Wu *et al.* 2010). Briefly, 100 μl serum was mixed with 300 μl H_2_O and 600 μl acetonitrile and placed for 30 min at 4 °C. After centrifuged at 12,000 *g* for 30 min at 4 °C, the supernatant was concentrated by vacuum centrifugation. The precipitate was redissolved with 100 μl H_2_O and processed to deglycosylation according to the instruction (NEB, USA). The deglycosylated proteins were redissolved with Sample buffer (125 mmol/L Tris Base, 20% glycerol, 4% SDS, 4% β-mercaptoethanol, and 0.04% bromophenol blue) with EDTA-free protease and phosphatase inhibitors (Thermo, USA). The protein samples were further denatured at 95 °C for 5 min.

### Colloidal blue staining and mass spectrometry detection

Serum protein samples were separated on 10% Tricine-gels and subjected to Colloidal blue staining (Life Technologies, USA) (Schagger 2006). The indicated bands were cut into slices for MS detection (Fig. 3B, C). In-gel digestion of every slice was performed as previously described (Chen *et al.* 2016). The resulting peptide mixtures were dried and stored at −80 °C until further LC–MS/MS analysis.

LC–MS/MS analysis of serum peptide mixtures was performed on a Q Exactive mass spectrometer with a nano-electrospray ion source (Thermo, USA) coupled with an EasyLC nano HPLC system. The digested peptides were then loaded onto a C18 trap column with an autosampler, eluted onto a C18 column (100 μm × 15 cm) packed with ReproSil-Pur 130 C18-AQ 3 μm particles (Dr. Maisch HPLC GmbH, Germany).

All MS/MS spectra were acquired in a data-dependent scan mode, where one full-MS scan was followed with ten MS/MS scans. The full-scan MS spectra (300–1600 *m*/*z*) were acquired with a resolution of 60,000 at *m*/*z* 400 after accumulation to a target value of 3e6. The 20 most abundant ions found in MS1 were selected for fragmentation at a normalized collision energy of 27% (Chen *et al.* 2016).

The LC–MS/MS data were searched against the homemade MMD using the Proteome Discoverer 1.4 with SEQUEST as search engine (Thermo, USA). Search parameters were set as follows: enzyme: trypsin; precursor ion mass tolerance: 10 ppm; fragment ion mass tolerance: 0.02 Da. The maximum number of miss-cleavages by trypsin was set as two for peptides. The variable modification was set to oxidation of methionine. The fixed modification was set to carboxyamidomethylation of cysteine.

### Signal peptide prediction

According to websites “http://phobius.sbc.su.se” and “http://www.cbs.dtu.dk/services/TargetP/,” Phobius and TargetP were used for the prediction of SEP signal peptide. The output format was based on TargetP. The SEPs were listed positively in the supplemental table only when both methods returned a positive signal peptide prediction. The final prediction was based on the scores on mTP, SP, and another. mTP was a mitochondrial targeting peptide. SP was a signal peptide for secretory pathway, which was shown as “S” in the supplemental table, and “–” was any other location. Reliability class (RC) contains five classes, in which “1” means the strongest prediction. TPlen showed the predicted presequence length.

### Conservation analyses

The corresponding nucleotide sequences for SEP3, SEP12, SEP33, and SEP54 ORFs were obtained from NCBI database (https://www.ncbi.nlm.nih.gov/), respectively, as reported previously (Lee *et al.* 2015). BLAST search was processed to ensure correct extraction of the nucleotide sequences. The protein sequences of six species, human (*Homo sapiens*), chimpanzee (*Pan troglodytes*), swine (*Homo sapiens*), dog (*Canis lupus familiaris*), rat (*Rattus norvegicus*), and mouse (*Mus musculus*), were aligned using Clustal Multiple Alignment.

### Immunoassay

The corresponding antigen peptide for SEPs was conjugated to Keyhole Limpet Hemocyanin (KLH) and injected into rabbits. The antigen information was listed here: RGRKFPQNAL for SEP3, SSKPIERSYMI for SEP12, RNKDAILEALRE for SEP33, and KAPEGAPSFGKA for SEP54. IgG purified sera were used for the detection of serum SEPs by Western blot.

For Western blot, serum protein samples were prepared in Sample buffer with EDTA-free protease and phosphatase inhibitors (Thermo, USA), heated at 95 °C for 5 min, ran on a 10% Tricine-gels and transferred to 0.4 μm PVDF membranes (Merck, Germany) at 100 mA for 30 min. Membranes were blocked with 5% nonfat dry milk for 1 h at room temperature (RT) and incubated with primary antibody (1:500–1:2,000 dilution) overnight at 4 °C, followed by secondary HRP-conjugated antibodies (1:10,000) for 1 h at RT. Chemiluminescence was detected and imaged using ECL (PerkinElmer Life Sciences, Waltham, MA).

### Statistical analyses

Data were presented as mean ± SEM unless specifically indicated. The statistical analyses were performed using GraphPad Prism 6. Comparisons of significance between groups were performed using Student *t* tests as indicated.

## Electronic supplementary material

Below is the link to the electronic supplementary material.

Supplementary material 1 (PDF 25 kb)

## References

[CR1] Anderson NL, Anderson NG (2002). The human plasma proteome: history, character, and diagnostic prospects. Mol Cell Proteomics.

[CR2] Anderson DM, Anderson KM, Chang CL, Makarewich CA, Nelson BR, McAnally JR, Kasaragod P, Shelton JM, Liou J, Bassel-Duby R, Olson EN (2015). A micropeptide encoded by a putative long noncoding RNA regulates muscle performance. Cell.

[CR3] Anderson DM, Makarewich CA, Anderson KM, Shelton JM, Bezprozvannaya S, Bassel-Duby R, Olson EN (2016). Widespread control of calcium signaling by a family of SERCA-inhibiting micropeptides. Sci Signal.

[CR4] Aramayo R, Polymenis M (2017). Ribosome profiling the cell cycle: lessons and challenges. Curr Genet.

[CR5] Bazzini AA, Johnstone TG, Christiano R, Mackowiak SD, Obermayer B, Fleming ES, Vejnar CE, Lee MT, Rajewsky N, Walther TC, Giraldez AJ (2014). Identification of small ORFs in vertebrates using ribosome footprinting and evolutionary conservation. EMBO J.

[CR6] Chen X, Xu S, Wei S, Deng Y, Li Y, Yang F, Liu P (2016). Comparative proteomic study of fatty acid-treated myoblasts reveals role of Cox-2 in palmitate-induced insulin resistance. Sci Rep.

[CR7] Chew GL, Pauli A, Rinn JL, Regev A, Schier AF, Valen E (2013). Ribosome profiling reveals resemblance between long non-coding RNAs and 5′ leaders of coding RNAs. Development.

[CR8] Chu Q, Ma J, Saghatelian A (2015). Identification and characterization of sORF-encoded polypeptides. Crit Rev Biochem Mol Biol.

[CR9] Cohen SM (2014). Everything old is new again: (linc)RNAs make proteins!. EMBO J.

[CR10] Derrien T, Johnson R, Bussotti G, Tanzer A, Djebali S, Tilgner H, Guernec G, Martin D, Merkel A, Knowles DG, Lagarde J, Veeravalli L, Ruan X, Ruan Y, Lassmann T, Carninci P, Brown JB, Lipovich L, Gonzalez JM, Thomas M, Davis CA, Shiekhattar R, Gingeras TR, Hubbard TJ, Notredame C, Harrow J, Guigo R (2012). The GENCODE v7 catalog of human long noncoding RNAs: analysis of their gene structure, evolution, and expression. Genome Res.

[CR11] D’Lima NG, Ma J, Winkler L, Chu Q, Loh KH, Corpuz EO, Budnik BA, Lykke-Andersen J, Saghatelian A, Slavoff SA (2017). A human microprotein that interacts with the mRNA decapping complex. Nat Chem Biol.

[CR12] Echan LA, Tang HY, Ali-Khan N, Lee K, Speicher DW (2005). Depletion of multiple high-abundance proteins improves protein profiling capacities of human serum and plasma. Proteomics.

[CR13] Hanada K, Higuchi-Takeuchi M, Okamoto M, Yoshizumi T, Shimizu M, Nakaminami K, Nishi R, Ohashi C, Iida K, Tanaka M, Horii Y, Kawashima M, Matsui K, Toyoda T, Shinozaki K, Seki M, Matsui M (2013). Small open reading frames associated with morphogenesis are hidden in plant genomes. Proc Natl Acad Sci USA.

[CR14] Housman G, Ulitsky I (2016). Methods for distinguishing between protein-coding and long noncoding RNAs and the elusive biological purpose of translation of long noncoding RNAs. Biochem Biophys Acta.

[CR15] Ingolia NT, Ghaemmaghami S, Newman JR, Weissman JS (2009). Genome-wide analysis *in vivo* of translation with nucleotide resolution using ribosome profiling. Science.

[CR16] Ingolia NT, Lareau LF, Weissman JS (2011). Ribosome profiling of mouse embryonic stem cells reveals the complexity and dynamics of mammalian proteomes. Cell.

[CR17] Ingolia NT, Brar GA, Stern-Ginossar N, Harris MS, Talhouarne GJ, Jackson SE, Wills MR, Weissman JS (2014). Ribosome profiling reveals pervasive translation outside of annotated protein-coding genes. Cell Rep.

[CR18] Jedrychowski MP, Wrann CD, Paulo JA, Gerber KK, Szpyt J, Robinson MM, Nair KS, Gygi SP, Spiegelman BM (2015). Detection and quantitation of circulating human irisin by tandem mass spectrometry. Cell Metab.

[CR19] Ji Z, Song R, Regev A, Struhl K (2015). Many lncRNAs, 5′UTRs, and pseudogenes are translated and some are likely to express functional proteins. eLife.

[CR20] Kay R, Barton C, Ratcliffe L, Matharoo-Ball B, Brown P, Roberts J, Teale P, Creaser C (2008). Enrichment of low molecular weight serum proteins using acetonitrile precipitation for mass spectrometry based proteomic analysis. Rapid Commun Mass Spectrom.

[CR21] Lee C, Zeng J, Drew BG, Sallam T, Martin-Montalvo A, Wan J, Kim SJ, Mehta H, Hevener AL, de Cabo R, Cohen P (2015). The mitochondrial-derived peptide MOTS-c promotes metabolic homeostasis and reduces obesity and insulin resistance. Cell Metab.

[CR22] Magny EG, Pueyo JI, Pearl FM, Cespedes MA, Niven JE, Bishop SA, Couso JP (2013). Conserved regulation of cardiac calcium uptake by peptides encoded in small open reading frames. Science.

[CR23] Makarewich CA, Olson EN (2017). Mining for micropeptides. Trends Cell Biol.

[CR24] Matsumoto A, Pasut A, Matsumoto M, Yamashita R, Fung J, Monteleone E, Saghatelian A, Nakayama KI, Clohessy JG, Pandolfi PP (2017). mTORC1 and muscle regeneration are regulated by the LINC00961-encoded SPAR polypeptide. Nature.

[CR25] Menschaert G, Van Criekinge W, Notelaers T, Koch A, Crappe J, Gevaert K, Van Damme P (2013). Deep proteome coverage based on ribosome profiling aids mass spectrometry-based protein and peptide discovery and provides evidence of alternative translation products and near-cognate translation initiation events. Mol Cell Proteomics.

[CR26] Nelson BR, Makarewich CA, Anderson DM, Winders BR, Troupes CD, Wu F, Reese AL, McAnally JR, Chen X, Kavalali ET, Cannon SC, Houser SR, Bassel-Duby R, Olson EN (2016). A peptide encoded by a transcript annotated as long noncoding RNA enhances SERCA activity in muscle. Science.

[CR27] Omenn GS (2007). The HUPO human plasma proteome project. Proteomics Clin Appl.

[CR28] Schagger H (2006). Tricine-SDS-PAGE. Nat Protoc.

[CR29] Smith JE, Alvarez-Dominguez JR, Kline N, Huynh NJ, Geisler S, Hu W, Coller J, Baker KE (2014). Translation of small open reading frames within unannotated RNA transcripts in *Saccharomyces cerevisiae*. Cell Rep.

[CR30] Tautz D (2009). Polycistronic peptide coding genes in eukaryotes—how widespread are they?. Brief Funct Genomics Proteomics.

[CR31] Ulitsky I, Shkumatava A, Jan CH, Sive H, Bartel DP (2011). Conserved function of lincRNAs in vertebrate embryonic development despite rapid sequence evolution. Cell.

[CR32] Wu J, An Y, Pu H, Shan Y, Ren X, An M, Wang Q, Wei S, Ji J (2010). Enrichment of serum low-molecular-weight proteins using C18 absorbent under urea/dithiothreitol denatured environment. Anal Biochem.

