# Supplementary material for: Identification of small ORF-encoded peptides in mouse serum
Source: Biophys Rep. 2018 Mar 8;4(1):39–49. doi: 10.1007/s41048-018-0048-0 (PMC5860097; doi:10.1007/s41048-018-0048-0)
Supplement: Supplementary file 1 — Supplementary material 1 (PDF 25 kb) [file 41048_2018_48_MOESM1_ESM.pdf]

Supplemental table

SEP NO.	Length	mTP	SP	Other	Localization	RC	TPlen
1	14	0.067	0.034	0.967	-	1	-
2	43	0.046	0.393	0.658	-	4	-
3	11	0.075	0.03	0.965	-	1	-
4	12	0.065	0.033	0.97	-	1	-
5	9	0.063	0.032	0.971	-	1	-
6	10	0.066	0.032	0.969	-	1	-
7	22	0.224	0.041	0.817	-	3	-
8	8	0.063	0.035	0.969	-	1	-
9	14	0.063	0.035	0.969	-	1	-
10	22	0.236	0.04	0.822	-	3	-
11	27	0.12	0.5	0.277	S	4	19
12	9	0.059	0.033	0.973	-	1	-
13	19	0.078	0.05	0.937	-	1	-
14	16	0.102	0.035	0.936	-	1	-
15	15	0.061	0.037	0.969	-	1	-
16	19	0.081	0.04	0.942	-	1	-
17	15	0.117	0.036	0.925	-	1	-
18	15	0.072	0.036	0.962	-	1	-
19	15	0.085	0.035	0.953	-	1	-
20	27	0.084	0.056	0.918	-	1	-
21	11	0.058	0.033	0.973	-	1	-
22	20	0.234	0.031	0.812	-	3	-
23	9	0.063	0.033	0.971	-	1	-
24	29	0.052	0.733	0.228	S	3	26
25	23	0.248	0.084	0.718	-	3	-
26	15	0.201	0.038	0.851	-	2	-
27	24	0.08	0.096	0.896	-	1	-
28	17	0.095	0.032	0.946	-	1	-
29	18	0.132	0.036	0.907	-	2	-
30	22	0.057	0.042	0.965	-	1	-
31	21	0.063	0.049	0.953	-	1	-
32	27	0.149	0.071	0.84	-	2	-
33	8	0.057	0.032	0.974	-	1	-
34	20	0.118	0.044	0.893	-	2	-
35	9	0.062	0.032	0.972	-	1	-
36	20	0.095	0.363	0.553	-	5	-
37	13	0.068	0.034	0.968	-	1	-
38	19	0.075	0.041	0.948	-	1	-
39	26	0.072	0.049	0.948	-	1	-
40	7	0.056	0.032	0.975	-	1	-
41	15	0.08	0.034	0.957	-	1	-
42	41	0.062	0.057	0.947	-	1	-
43	8	0.061	0.031	0.972	-	1	-
44	21	0.1	0.045	0.928	-	1	-
45	9	0.058	0.033	0.974	-	1	-
46	21	0.068	0.038	0.958	-	1	-
47	10	0.059	0.033	0.972	-	1	-
48	12	0.07	0.032	0.967	-	1	-
49	6	0.059	0.03	0.974	-	1	-
50	21	0.118	0.035	0.915	-	2	-
51	9	0.06	0.033	0.973	-	1	-
52	23	0.045	0.638	0.494	S	5	22
53	18	0.067	0.047	0.952	-	1	-
54	8	0.058	0.032	0.974	-	1	-
